# Parental experience of whole genome sequencing for children with sensorineural hearing loss

**DOI:** 10.1080/17482631.2026.2641802

**Published:** 2026-03-12

**Authors:** Johanna Elander, Maria Värendh, Johannes K. Ehinger, Karin Stenfeldt, Stephen Widén

**Affiliations:** a Otorhinolaryngology, Head and Neck Surgery, Department of Clinical Sciences Lund, Lund University, Lund, Sweden; b Department of Otorhinolaryngology, Skåne University Hospital, Lund, Sweden; c School of Medical Sciences, Faculty of Medicine and Health, Örebro University, Örebro, Sweden; d Department of Otorhinolaryngology, Örebro University, Örebro, Sweden; e Logopedics, Phoniatrics and Audiology, Department of Clinical Sciences Lund, Lund University, Lund, Sweden; f Audiological Research Centre, Faculty of Medicine and Health, Örebro University, Örebro, Sweden

**Keywords:** Sensorineural hearing loss, genetic sequencing, parental experiences, thematic analysis, interview study

## Abstract

**Purpose:**

The purpose of this in-depth interview study was to explore how parents of children with sensorineural hearing loss (SNHL) experienced genetic testing and whether they experienced risks and benefits.

**Background:**

Most children with SNHL have a genetic etiology, which can be identified through genetic sequencing. A genetic test does not influence treatment, and whether patients and parents perceived genetic tests as valuable is unclear.

**Methods:**

In this study, 10 parents of children with SNHL who underwent genetic testing were interviewed, and the content was analyzed using inductive thematic analysis.

**Results:**

Three global themes were identified. In the first theme, (1) *Limited knowledge creates uncertainty*, parents described uncertainty related to the information provided, the test result itself and child-related factors. The second theme, (2) *Genetic knowledge is considered important for the family and the future*, explored the importance of knowledge. Parents wanted an explanation to make the future predictable, and the test had practical implications. In the last category, (3) *Knowledge adds complexity and can be challenging*, ethical considerations and risks associated with knowledge were highlighted.

**Conclusion:**

The main conclusion was that parents experienced that genetic testing provided valuable personal information and had practical implications. However, a genetic diagnosis can cause concern and may affect family planning.

## Background

In recent decades, genome sequencing has become available for genetic diagnostics in patients with sensorineural hearing loss (SNHL). Within the HearSeq project, DNA from 96 patients was sequenced (11 with whole exome sequencing (WES) and 85 with whole genome sequencing (WGS)) (Elander et al., 2022, 2025). A panel with 178–210 genes (Clinical-Genomics-Lund, 2024) (the panel was updated regularly) associated with SNHL was applied.

SNHL is the most frequent sensory deficiency affecting one to two of every thousand infants (Morton & Nance, 2006). There is a detectable genetic cause in more than half of the children with prelingual SNHL (<2 years)(Boudewyns et al., 2023; Mitchell & Morton, 2021). In the HearSeq cohort, 52 of the 85 patients examined with WGS had prelingual moderate to profound SNHL, and a genetic cause was identified in 60% (*n* = 31) of those patients.

In cases without a definite genetic diagnosis or other defined aetiology, a genetic cause still cannot be excluded. Furthermore, the symptomatology of detected genetic variants is not always fully understood, and the penetrance of symptoms may vary. In addition, structural variants (e.g., deletions, duplications, translocations) not related to SNHL may sometimes be detected, despite the application of the SNHL-specific gene panel. Genetic variants related to isolated SNHL predominate, whereas syndromic SNHL is expected in approximately 30% of cases with a genetic diagnosis (Alford et al., 2014).

Persons with SNHL are treated with hearing aids (HA) or cochlear implants (CI) and additional communication devices depending on the severity of the hearing loss. Additionally, hearing education interventions, as well as sign language, are essential. Although a clinical gene therapy trial for pathological variants in one gene (*OTOF*) has been reported to be successful (Hu et al., 2024; Lv et al., 2024; Qi et al., 2024), there is currently no genetic therapy available for clinical use to treat SNHL.

From a medical point of view, there are advantages in obtaining a genetic diagnosis, both in terms of prognostic factors and for the early detection of other symptoms related to hearing loss, i.e., early diagnosis of syndromic hearing loss (Korver et al., 2017; Liming et al., 2016; Mitchell & Morton, 2021; Sloan-Heggen et al., 2016). However, a genetic test does not affect treatment. This raises the question of the importance of testing and whether testing adds value for patients and their families. Personal utility is separated from other ways of evaluating genetic tests, where analytic validity, clinical validity, clinical utility (improvement of health outcome) and ethical, legal, and social issues have been the main focus (Sanderson et al., 2005). Bunnik et al. (2015) made an interesting point by arguing that, to gain personal utility, genetic tests must contain both meaningful information and have a purpose, i.e., both clinical validity and utility need to be assessed. Personal utility is closely linked to patient-centred medicine, a holistic approach embracing personal context, values, and needs. An approach that results in optimisation of the clinical outcome (Ekman et al., 2011; Gluyas, 2015). In this study, the patient-centred perspective was represented by the parental experience of genetic testing.

An Australian study from 2021 (Tutty et al., 2021) aimed to generate an understanding of the personal utility of WES in children with SNHL. It was an inductive content analysis of text from open-ended response questionnaires completed by 67 parents. Among other things, they concluded that the testing led to a sense of control and empowerment and was a way to avoid unpleasant surprises in the future.

In a systematic review article from 2017, including 27 studies, the personal utility of genetic sequencing in general was analysed (Kohler et al., 2017). They concluded that personal utility could be identified at both personal (affective, cognitive, and behavioural) and a social level. Based on the results of the review study, the same research team developed a personal utility (PrU) scale as a tool to measure the personal benefits of genetic testing. For parents of genetically tested children, three key factors were identified: benefits for the child, affective parental benefits, and parental control (Turbitt et al., 2024).

In the literature, the focus has been on optimising the diagnostic procedure and describing the diagnostic yield with sequencing techniques. However, patient and family perceptions of genetic testing and diagnosis have become increasingly important as medicine becomes more person-centred. As caregivers, parents are the most appropriate representatives of the child, and their views are therefore important to consider. Parental experience and perceived utility of genetic sequencing in children with diverse symptomatology, have been described in recent studies (Halley et al., 2022; Hayeems et al., 2021; Marathe et al., 2024). However, parental experience of genetic sequencing in relation to SNHL needs further exploration.

The aim of this in-depth interview study, using an inductive thematic approach, was to explore how parents of children with SNHL experience genetic testing and the test result. In addition, the experienced risks and benefits of genetic testing (WGS) were penetrated.

## Method

This study was approved by the Swedish Ethical Review Authority (Dnr 2022-06149-02 and 2018/282), and after oral and written information, all participating parents signed a consent form.

### Study design

**Figure 1.**
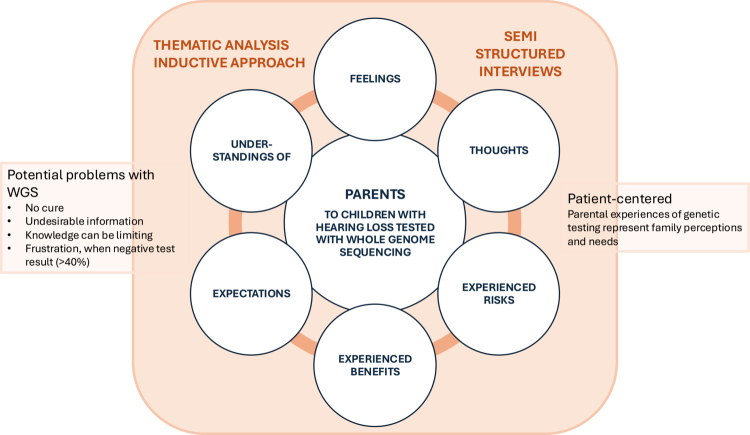
Conceptual framework of the study. Parents of WGS-tested children with SNHL were interviewed. The data were coded and analysed using thematic analysis to explore how parents experienced the genetic testing.

A constructivist approach for analysing the parental experience of a phenomenon, genetic testing in children with SNHL, was needed. In this qualitative study, thematic analysis was suitable for identifying experienced risks and benefits of genetic testing in children with SNHL. An inductive approach, where the analysis is grounded in the data itself without a preexisting theory, was used (Attride-Stirling, 2001; Kiger & Varpio, 2020; Varpio et al., 2020). The data were coded and categorised in a stepwise (Attride-Stirling, 2001; Braun & Clarke, 2006), reflexive way (Braun & Clarke, 2021) by two researchers. In addition to coding and identifying themes, the process included constructing, describing, exploring, and summarising networks and interpreting patterns (Attride-Stirling, 2001). Patterns and themes related to the research question were identified. The studied phenomenon was the parental experience of genetic testing in a child. In this study, genetic testing relates to the process from pretest information and follow-up after the test result. The inductive approach was consistent with the aims of exploring subjective experiences without a prior theoretical model, and the conceptual framework was followed throughout the study (Figure 1).

### Sampling and recruitment

The interviews were conducted from February to May 2023. The inclusion criteria were parents of children under 5 years of age with bilateral SNHL who had undergone genetic sequencing. To ensure semantic consistency, the ability to communicate in spoken Swedish was mandatory.

Parents of the 20 genetically sequenced children, under 5 years of age, who had last undergone genetic testing at the University hospitals in Lund and Örebro, were asked by letter to participate in the study and were then contacted via a phone call. Parents of nine of the children were willing to participate in an interview and provided written informed consent (Table I). All but one interview was conducted with a single parent at the time. Most interviews lasted 35–45 min. The outliers were two interviews, where one lasted 1 h and 5 min and the other lasted 20 min. The time span from taking the genetic test to conducting the interview ranged from 7 to 41 months, with a median of 16 months.

**Table I.** Ten participating parents to children with sensorineural hearing loss (SNHL) examined with whole genome sequencing.

Participant	1	2	3	4	5	6	7	8	9
Sex	Male	Female	Female	Female	Male/Female	Female	Male	Female	Female
Degree of SNHL of the child	Profound	Moderate	Mild	Profound	Profound	Mild	Profound	Profound	Profound
Genetic diagnosis	None	None	None	None	Isolated SNHL	Isolated SNHL	Pendred syndrome	Wardenburg syndrome	Usher syndrome

Later, an additional four parents were asked to participate, one of whom, not a native Swedish speaker, chose to participate. In this case, Swedish was complemented with explanations in English, the second language for both the interviewer and the parent. No obvious new themes were identified. The interview was later excluded because it did not meet the inclusion criteria of being conducted in Swedish.

### Interview process

Semi-structured interviews with ten parents (three fathers and seven mothers) were conducted by the same interviewer. The parents received a consecutive recording numbers (1–9), and their identities were anonymized in accordance with the ethical approval.

An interview guide was developed by the authors JE and SW to explore themes related to the purpose of the study. When the interviews were conducted, this interview guide was used. The guide covered eight topics, including questions about the information process, expectations, feelings, experienced risks and benefits before and after testing, and ethical considerations (supplement 1). The interviewer added follow-up questions related to the answers of the parents during the interviews to explore the experiences of the parents. Four of the interviews were conducted in a meeting room at the research unit at the Department of Audiology in Lund or Örebro, two were conducted in the home of the participants, while three interviews were conducted on the digital platform Zoom. All interviews were recorded. The interviewer (JE) proceeded with verbatim manual transcription of all the interviews and started the analytic process during the relistening of the interviews.

### Analytic process

All the material was re-read and coded by the interviewer. Without a preexisting codebook, the interviews were also read and coded by a senior researcher (SW). The codes were discussed until there was an agreement between the two researchers. The interrelated codes were clustered together and organised into basic themes. Following the inductive approach, and working from the empirical data, the basic themes were organised into organising themes. From the organising themes, the global themes were identified, as a condensation of the concepts from the lower levels, as described by Attride-Stirling (2001) and Skovdal (2015). This is similar to the stepwise thematic analysis process described by Braun and Clarke (2006). They also describe potential pitfalls in the analysis process, and efforts have been made to avoid these pitfalls (Braun & Clarke, 2021). For example, the pitfall “confusing themes and topics” was identified when reviewing the themes and, hence, the themes were redefined. The quotes were analysed, and the essence of the content formed the basic themes. Sometimes quotes that at first seemed contradictory were interpreted as belonging to the same basic theme (see example in Results section “Relief at both what was found and what was not found”). Other statements needed to be separated as they illustrated variations in reasoning. The basic themes formed the basis of the organising themes, which were then interpreted as belonging to a global theme. The codes and themes were compared, discussed, and reassessed during the analytic process. There was consistency in coding and coherence in the deciphered themes. During the last interview (and the excluded interview conducted in English), no obvious new themes were generated, and thus no further participants were included.

### Illustration of the analytic process

Table II describes the analytical process for one of the three global themes, “1. Limited knowledge creates uncertainty”. In the first column, the organising themes are presented, followed by the basic themes and example of the codes (quotes).

**Table II.** Illustration of the first global theme.

1. Limited knowledge creates uncertainty		
Organising themes	Basic themes	Quotes
1.1 Parents need for information was not being met	Wish for written information	*“Yes, I would need to have everything in writing.”* (# 6)
	Lack of communication	A mother receiving genetic test results about her child with Usher syndrome without any preparation. *“It was horrible. It was horrible. Because we didn't even know about it. We got a summons to the ENT specialist and that's about the only thing I can be really angry about, or not angry, but I don't know how they could have done it any better either. We got a piece of paper, and we thought we'll go to the ENT doctor and make a regular visit. We didn't know, it didn't say anything. There was nothing about, well, about genetic testing or anything in the paper.”* (# 9)
	Insufficient information	*“We didn't get a lot of information really, in general, neither about syndromes nor this genetic test, but the only thing we really got was that you do a genetic test to rule out that you have a syndrome. That's what we got really”* (# 9)
	No reassurance without genetic information	A parent who wanted to take a genetic test, but the doctor hesitated and delayed the genetic testing. *“It was like this… you don't have to worry, all the time.”* (# 2)
1.2 Inconclusive test results were stressful	Test result is not reliable	*“Whatever it is, if someone tells you that it's not one hundred per cent reliable, you still rely on it. Well, okay, he's got nothing, so we can rest easy, and if it turns out later that there is something, then that's where the risk lies.”* (# 9)
	Vague test results cause concerns	*“It's these grey zone cases, that's it, it's hard not to get a clear answer.”* (# 6)
1.3 Uncertainty if genetic testing is in the best interest of the child	Child function and genetics do not harmonise	*“And then if you already know the genetic information about someone like that. Because it doesn't say much, it says he's deaf, but it doesn't say he has two functioning cochlear implants and sign language, signs with support and…”* (# 8)
	The test is not used in the best interest of the child	*“That it falls into the wrong hands. That's the risk, and I feel that the world is not so risk-free anymore. That you should not be completely naive. That's what it is.”* (# 4)
	The child´s opinion about genetic testing is unknown	“*That is if you believe that he, we chose to find out everything about him and his genetics. Right now, as his parents. But maybe he doesn't want to know why. Maybe he just accepts that this is the way it is and doesn't want to know more.”* (# 8)

### Translation

For the translation of the quotes from Swedish into English, the AI translator DeepL (VAT-ID: DE349242045) was used, and the content of each quote was manually checked by JE and re-translated to ensure that the meaningful content was intact and unchanged.

## Results

**Figure 2.**
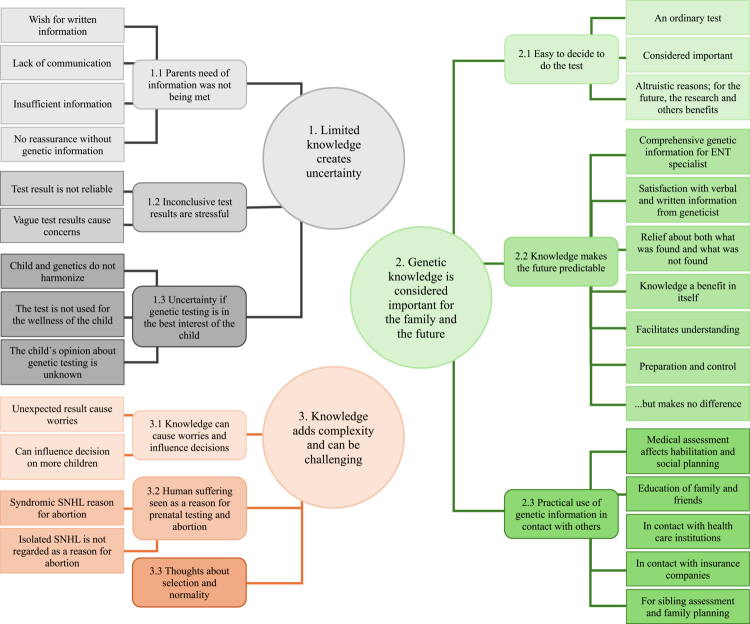
Three themes related to parental experience with WGS were identified: “Limited knowledge creates uncertainty”, “Genetic knowledge is considered important for the family and the future” and “Knowledge adds complexity and can be challenging”.

From the transcribed and coded interviews, three global themes were identified: “Limited knowledge creates uncertainty”, “Genetic knowledge is considered important for the family and the future” and “Genetic knowledge adds complexity and can be challenging” (Figure 2). All global themes are in different ways related to knowledge. The longing for an answer and how both knowledge and uncertainty influence the experience of the genetic test. Knowledge can also lead to more questions and produce outcomes apart from the primary aim of the genetic test. Three main categories were identified in each theme, which, in most cases were based on several sub-categories.

Theme 1. Limited knowledge creates uncertainty

Parental experience of uncertainty centred on the information provided, the test result itself and factors related to the child.

**1.1 Parents need of information was not met**

Some parents experienced a gap between the parental need for information and the received information. Recognition of parental needs and expectations is crucial for the information process. There was a *wish for written information*, as one mother expressed as following:

*“Absolutely, you would like to have a written answer and maybe a little bit based on what we have discussed or said.”* (# 4).

Additionally, there were parents who felt misinformed by their treating physician, mainly due to a *lack of communication*, both between the parent and the treating physician and between physicians with different specialist competences. The lack of communication was troublesome, as exemplified in a case where the information process was disrupted by misunderstandings, long waiting times, and frustration. When doctors with different specialties interpreted the genetic results differently, one said that Usher syndrome was excluded, and the other ordered check-ups, the parents experienced that it was hard to know who to trust. Additionally, parents noted that they received *insufficient information.* Others experienced that offers from the Department of Clinical Genetics were not being transferred to the family. This resulted in feelings of being withheld the opportunities to be properly informed about the condition of their child.

*“We have received these test results from there* [clinical genetics] *and it also says that if the family wishes and wants, they can be referred to clinical genetics. It says in the referral response from there. And we didn't do that, we didn't get that, that offer.”* (# 6).

In cases where the treating physician tried to be reassuring before the genetic test was taken or before the test result was obtained, this was experienced as counterproductive and *not reassuring without genetic information.* This was expressed as feelings of frustration and that their concerns were not taken seriously.

**1.2 Inconclusive test results are stressful**

In this second category, the limited knowledge is related to the test result itself and the built-in knowledge restriction; that there is still more to understand about genotypes related to hearing loss.

Some parents acknowledged the risk that *the test was not completely reliable* and that this was something that could be troublesome.

*“Well, okay, he's got nothing, so we can rest easy, and if it turns out later that there is something, then that's where the risk lies.”* (# 9).

Another stressful situation was to get a *vague test result*. For example, one child had a pathogenic variant in a gene where the phenotype could be either isolated hearing loss or Usher syndrome. This ambiguous result, and the offered eye examinations were stressful for the parents and not aware what to expect was difficult to manage.

*“I have read quite a lot about the gene and so on and see that there is a grey area and see that it's not just black and white, it's not just either or, but that there are people with this gene who get an atypical Usher, which is a bit more like Usher type 3*.” (# 6).

**1.3 Uncertainty if genetic testing is in the best interest of the child**

In this subcategory, knowledge and uncertainties related to the child are in focus.

The genetic result might *not harmonise with the functions of the child*. Parents acknowledge that the genetic code can indicate that someone has become deaf, has visual problems or has a rare disease. However, they experienced a risk that the perception of the disease by others, may not be in line with the personality or the functional resources of the child, treated with cochlear implants, or other habilitation aids. Another potential risk identified was that the genetic data could be used for *purposes other than the best interest of the child*; it is not known how this will be regulated in the future. Depending on legislation and rules regulating genetic data, the test could be misused, and one mother expressed it like this:

*“That it falls into the wrong hands. That's the risk, and I feel that the world is not so risk-free anymore. That you should not be completely naive.”* (# 4).

Also, there is an *uncertainty if the child when he/she grows up favours the genetic information*. Even if the guardians did what they thought was in the best interest for their child, they decided to take the test without knowing the future wishes of the child.

*“But maybe he doesn't want to know why. Maybe he just accepts that this is the way it is and doesn't want to know more.*” (# 8).

Theme 2: Genetic knowledge is considered important for the family and the future

This theme covers parental thoughts and opinions on the usefulness of genetic information. Knowledge tends to be the central subject. An urge after knowing why, to get an explanation of what this means for me and what it can lead to in the future is something all the participants experienced, one way or another. The first category is linked to the decision to perform the genetic test, while the subsequent categories are related to the benefits of comprehensive knowledge for making the future predictable and practical consequences of a confirmed diagnosis.

**2.1 Easy to decide to do the test**

For the parents, the decision to have the child undergo a genetic test was in most cases not a difficult decision to make, and it was seen as *an ordinary test*, a blood test.

*“For us it was more, leave sample, blood test. Prick in the arm and they are done, then we wait for a letter in the post. There was not much more.”* (# 7).

The genetic test was *considered important* and several participants said that they wanted to know why their child had SNHL.

*“We wanted to know what the cause was, and it was the least we could do, to leave some samples and see what the result is.”* (# 7).

Some acknowledged that the genetic test could reveal an unfavourable genetic finding. However, this was not a reason for not performing the test; rather, it was the opposite. The parents noted that if the genetic test revealed any comorbidity, they wanted to have the chance to find out as much as possible to be able to be prepared for future events. Most of the parents also experienced that they wanted to contribute to knowledge in this field and be part of the research, which can be characterised as an *altruistic standpoint*. The genetic testing was not merely done for their own child, but also for future children and the expanding genetic mapping.

*“Even if it can't help us or our children, or… maybe it can help someone else who suffers the same in the future.”* (# 3).

**2.2 Knowledge makes the future predictable**

Knowledge is important, not merely for medical reasons, but also parents described knowledge as the opposite of uncertainty, which made the future predictable. The first prerequisite was comprehensive information. Subsequently, parents experienced that the knowledge was related to favourable mental factors, leading to an understanding that provided a sense of control and of relief. The genetic information was thus used as a tool to manage the situation of having a child with hearing loss more adequately.

Some of the parents experienced that they received *comprehensive genetic information from the ENT specialist*, while others expressed *satisfaction with the information from the clinical geneticist*. In fact, none of the participants articulated dissatisfaction with the consultation at clinical genetics. One father expressed the following:

*“We went through, in detail, like what it's about, what symptoms you can get and what it looks like in the inner ear and etcetera, etcetera. So we got great information. We have all the information.”* (# 7).

An answer could also be experienced as *a relief, both about what was found and what was not found*. This means that even an answer without a genetic finding can be a relief. At least you find out what it is not. This may seem contradictory at first, as a genetic finding, but also a non-finding had similar outcomes. Some parents experienced relief that it was not Usher syndrome. Parents of a child who received a genetic diagnosis confirming isolated hearing loss, where the mother had worried that it was somehow her fault, also expressed relief.

*“It was really just a relief that had been released from the shoulders that, well, that you found out how it had happened. Instead of just walking there in your mind without knowing why it has happened.”* (# 5).

A father who had received a genetic result confirming a syndromic diagnosis, Pendred syndrome, felt relieved to obtain an answer. Although he admitted that he was sad about receiving the diagnosis, it helped him take measures for the future.

Furthermore, parents experienced that genetic *knowledge can be beneficial in itself and is not just limited to factors that adjust or improve assessment and treatment. Parents said that they wanted to know*. The knowledge seemed to be more important than the risk of receiving unwanted information.

*“I want to know, even if I get a very sad message, I still want to know”* (# 9).

To get *an answer facilitates understanding*, and other suspected reasons for SNHL can be excluded.

*“If we had still been walking around suspecting that there was something wrong with the birth, we might have felt worse, or very bad about it.”* (# 7).

Parents experienced that by understanding the genetic condition, they were able to explain to their children why they had a hearing loss and thus facilitating the understanding of the child as well. This was an issue also recognised by a mother with a negative genetic test.

*“It would have felt good to be able to tell my child. Explain to her why she has it, so that she had answers. It would have been easier to understand it”* (# 3).

A positive genetic test result provided an opportunity for *mental preparation, which led to a sense of control*.

*“We think it's comforting to know like what we can expect, what problems X might have in the future, what can we help him with” (# 8).*

Additionally, parents pointed out that the genetic information can be used in preparation. Even if the parents know that *the genetic information do not affect treatment*, they favour genetic information to be able to be prepared.

*“It doesn't really change anything. You love your child anyway, it's more because you want to be prepared”* (# 2).

**2.3 Practical use of genetic information in contact with others**

In addition, the participants in the study described that genetic knowledge had practical implications and was considered important as a guide for habilitation, social and family planning. It is also used as an educational tool in social interactions with family and friends, as well as in contact with health care or other institutions.

Knowledge of associated morbidity was considered important, as was the prognosis of the hearing loss. *Medical assessment affected habilitation and social planning*, e.g., the need for special education or the need for additional communication skills, such as sign language.

*“I mean just such a simple thing like we live in Örebro county; we have Sweden's largest School for the deaf and blind. Imagine if we had planned to move away from here and then we find out when he is ten years old that he is starting to go blind.” (# 9).*

A definite genetic diagnosis can also be an *educational aid in social interactions* and in managing speculation among *family and friends*.

“*Now I am talking about close family and friends of ours who, when we told them that our daughter has hearing loss, many of them started floating away in their thoughts and reading on the internet without knowing anything. But getting this answer has helped us a lot and put some stop to the speculation going on around us. Because it hasn't been easy to hear others speculate about our child.”* (# 7).

Knowledge can be used *in contact with health care institutions* and to educate health professionals. One participant described how they always ended up with an ENT specialist when they went to the emergency room, even if they experienced diarrhoea and fever, only because the child had cochlear implants. The genetic knowledge of the condition of the child, Waardenburg syndrome, and what it entails, helped the parents to guide the health professionals when searching for health care for other reasons. The same family also had difficulty switching child insurance. In this case, they were able to use the genetic knowledge *in contact with the insurance company*, to claim that there were no other medical concerns in their child other than SNHL.

The last identified practical beneficial factor was related to *sibling assessment and family planning*, the usefulness of knowledge of inheritance patterns. One mother with a child with a *de novo* variant expressed it like this*…*

*“Because it affected, well, whether you would have more children or not. So that also became a thing. We* [the parents] *also got to be part of the result. Even though we originally did it for X's sake, we also got something out of it.”* (# 8).

Theme 3: Knowledge adds complexity and can be challenging

Risks connected to the knowledge and related ethical considerations were acknowledged by the parents. They realised that a genetic test result could give rise to worries about the child and affect family planning. Furthermore, genetic knowledge has raised ethical concerns. The parents expressed that there is relation between the severity of a condition and the relevance of prenatal diagnostics and abortion.

**3.1 Knowledge can cause worries and influence decisions**

The parents experienced that an *unexpected result caused worries* and was hard to manage. One mother expressed that even though she was well informed about possible genetic findings, it was difficult to obtain an unexpected Usher diagnosis.

On the other hand, parents also identified that there is a risk of worrying in advance or unnecessarily.

*“I can imagine that there may be things that you might not want to know if it is not possible to do anything about it. If you have a greater risk of getting certain diseases or so, it may not always be fun to know. However, it's good if you can do something about it and detect it early. But that's the risk you take, to worry unnecessarily.”* (# 3).

In addition, SNHL and a genetically identified cause *could influence decisions of having more children*. The participants regarded the use of genetic information for family planning and selection purposes as ethical.

*“We learnt that it was recessive, and all our children have a 25% risk of getting both mutations. Then we felt that we shouldn't have any more children and if I had become pregnant, we would have chosen to have an abortion.* (# 6).

**3.2 Human suffering seen as a reason for prenatal testing and abortion**

The parents experienced that SNHL is a manageable disability with hearing aids, cochlear implants, and other habilitative interventions. In cases of syndromic SNHL, they reported that human suffering for the parent, child and even society could be a reason to refrain from having children.

*“Let's take that as an example, if I had known that, and say she had Usher syndrome, I would not have had her. I can say that one hundred per cent.”* (# 1).

The participants described that *syndromic SNHL with multiple disabilities is a reason for abortion*, not only for the sake of the family, but also for the unborn child. They expressed that a life with multiple disabilities might not be the life you wish for someone. One parent explained that testing and abortion are not only for the sake of the child but also for the benefit of society.

*“I mean, because it's a burden, even though X is our child, we love him more than anything, but it's still a burden on both the healthcare system and his future as well.”* (# 9).

When it came to *isolated SNHL, this was not seen as a reason for prenatal diagnostics or abortion* to any large extent.

*“I don't think we would have rejected a child if we had been told it was just an isolated hearing loss. We would never have done that.”* (# 6).

**3.3 Thoughts about selection and normality**

To do prenatal testing and make decisions about what to do with the information is considered to be personal, since the parents experience that it does not affect anyone else. Still, notions of normality are actualised when genetic tests are carried out and parents could see the risk that traits are deselected or seen as not wanted in society.

*“But this is a big issue with ethics, because it involves selecting what is normal and what is not normal. And that is very difficult. Very, very difficult.”* (# 6).

Finally, a mother, with twins summoned up that it is hard to regret children who are already been born.

*“They have a great life, and they are great boys. So based on what has been when they were born and what is now, that's it. You can't opt out of that.”* (# 4).

## Discussion

Genetic variation and pathological findings in children with SNHL have been of great interest from a medical point of view (Boudewyns et al., 2023; Downie et al., 2020; Mehta et al., 2016; Nishio & Usami, 2015; Sloan-Heggen et al., 2016; Tropitzsch et al., 2022; Zazo Seco et al., 2017) and can guide the physician in further medical assessment when syndromic hearing loss is suspected or diagnosed. However, the lack of available gene therapies raises the question of whether genetic diagnosis adds value for families with children with SNHL. This interview study aimed to explore how the parents of children with SNHL experienced genetic testing and whether they identified risks with, or benefits from, performing the test.

### Main results

An important finding was that parents experienced that genetic testing provided valuable information on a personal level and had practical implications. However, it could be troublesome when the result was unclear or reliable.

The three global themes were all related to knowledge about the genetic aetiology of SNHL. Limited knowledge created uncertainty, whereas knowledge was considered important for the family both now and in the future. Knowledge could also add complexity and be challenging to handle.

While straightforward information could make the future predictable and have positive practical implications, the opposite was stressful and could cause concerns linked to future decisions and uncertainty about what was in the best interest for the child and the family.

The genetic knowledge was considered important, and as soon as there was a definite answer, the parents got a closure and were able to continue making plans for the future. In contrast, parents with an inconclusive answer seemed to be trapped in the information process, and some had difficulties to cope with uncertainty. Additionally, some parents may need additional information in writing or a referral to a clinical geneticist.

Parents had straightforward opinions regarding decisions related to family planning and ethical considerations. Although these decisions are complex, they do not appear to be ambiguous and are likely to be based on fundamental personal values.

### Result discussion

#### Uncertainty

Limited knowledge, by not receiving all the information or not understanding the test results, as well as the inherent uncertainty of the test or child-related issues, can create uncertainty, as described in the first theme (1. Limited knowledge creates uncertainty). Uncertainty is related to anxiety, a response to a potential threat (Blanchard & Canteras, 2024; Grupe & Nitschke, 2013). A potential threat can be as hard to handle as a complicated medical condition. For example, Ginsburg et al. (2023) studied the impact of an inconclusive screening sequencing test for cystic fibrosis in infants on their mothers. They concluded that mothers of children without symptoms, but with a variant of uncertain significance (VUS), suffered from anxiety and depression to the same extent as mothers of children with the disease do (Ginsburg et al., 2023). Therefore, when genetic tests are performed on children with SNHL, uncertainty must be reduced and managed at an individual level. It is impossible to know in advance how parents will react to the results, and which parents will have difficulties comprehending the results.

Information to the parents can be provided in writing and, when needed, complemented with information from a clinical geneticist. In this way, the uncertainties identified in the first category “Parents need of information was not being met (1.1)” can be handled. This is probably most important in cases with inherent uncertainties related to the test result. In an era of expanding knowledge in the field of genetics, finding VUS or unexpected pathological findings will continue. The inherent risk of uncertainty with genetic testing is something that needs to be discussed with parents beforehand. To reduce uncertain test results, an argument is to refrain from testing or having fewer genes in our gene panels. The problem is that without testing, parents remain unsure or uncertain of why their child has SNHL. If you do the testing, the challenge is to explain that a genetic test is not always black or white. Moreover, there may be unexpected test results where the possibility to predict the future is limited. As one interviewee pointed out, there is also uncertainty about how legislation will regulate genetic data in the future and that the data may be used for purposes other than the well-being of the tested child.

To ensure that someone want the information in writing, there is an urge to obtain a deeper understanding of the condition or that would benefit from consulting a clinical geneticist were interpreted as success factors to reduce uncertainty based on the parental experiences.

#### Knowledge

Our second theme can be discussed using the context of coherence theory, developed by Aaron Antonovsky more than 40 years ago (Antonovsky, 1987). He reasoned that the ability to manage difficult situations (in this case, having a child with functional impairment) was connected to three elements: comprehensibility, manageability, and meaningfulness. In this context, the knowledge of why a child has SNHL provides comprehensibility. With a known disease-causing genetic variant, speculations about other causes can be avoided. When a known variant is accompanied by the expected phenotype, the condition is understandable, and the future may become more predictable (2.2). An understandable or cognitively comprehensible condition can become manageable. Manageability is a strong driving force for parents, as they strive to do what is best for their children. For this purpose, genetic testing and knowledge are valuable building blocks for parents helping them comprehend and manage the situation.

The first category, “easy to decide to do the test” (2.1), is a way to reach comprehensibility, but also to create meaningfulness by contributing to the research and helping other children. The use of genetic information in contact with others (2.3) is another way of using the knowledge for something meaningful.

This finding is in congruence with the Australian study (Tutty et al., 2021) about personal utility (with WES in children with SNHL), where a genetic test was related to a sense of control and empowerment. The reason for this is probably that knowledge can be anchored in reality, and the world becomes understandable. Dumez et al. argued that recognising patient knowledge and understanding that this knowledge can be differentiated in nature is fundamental to creating value from knowledge (Dumez & L’Espérance, 2024). This broader definition of the nature of knowledge is also appealing to this study.

#### Ethical considerations

The third theme is closely linked to ethical considerations. Parents identified that there is a risk of selection when introducing genetic testing that is useful for prenatal diagnostics and abortion. However, whether it is a true risk or a desirable consequence of testing is a subject where there can be different opinions. The connection between genetic testing and potentially complex standpoints and decisions regarding future children did seem surprisingly uncomplicated for the parents. These may be decisions that parents have reflected on more deeply, that are part of personal values and that are therefore, despite their complexity, easy to make. The sociocultural context, including cultural and religious beliefs, family structure, etc., can also influence decision-making on these issues.

Nevertheless, feelings and thoughts about future children did not seem to affect feelings for the present child. Whether parent–infant attachment is affected by a genetic test is rarely mentioned in the literature. However, obtaining an early diagnosis of hearing loss/deafness (established through the newborn screening programme) has been found to be appreciated by parents (Magnuson & Hergils, 1999, 2000). These experiences may be transferable to receiving an early genetic diagnosis of SNHL.

#### Utility and comparison with earlier studies

The utility of genetic sequencing in rare diseases can be viewed from different angles. While Hayeems et al. (Hayeems et al., 2022) studied ways to measure clinical utility, others concentrated on the patient or parental perspective of perceived utility. The themes of personal utility identified by Kohler et al. (2017) are related to the categories and subcategories identified in our study. For example, *affective personal benefits* can be identified in both category 2.1, where knowledge was considered important, and category 2.2, where it was related to satisfaction and relief. *Cognitive benefits* can be considered facilitating understanding (2.2), leading to mental preparation and control (2.2), while *behavioural benefits* can have practical implications for medical assessment, habilitation and family planning (2.3). *Social benefit* was seen in both category 2.1 (altruistic reasons, for the future, for the research and to benefit others) and in category 2.3, where it was more related to practical consequences in social contacts. Also, the themes identified with the parental personal utility scale (parental PrU) by Turbitt and Kohler et al. (2024), i.e., child benefits, affective parent benefits and parental control, had similarities with our results. Nevertheless, both clinical and personal utility can be regarded as important for parents. Smith et al. (2022) described five different utility domains: clinical, emotional, behavioural, cognitive and social utility. In this study too, there seems to be a congruence of themes, although with slightly different focus, interpretation, and evaluation. Compared to our study, similar content is described in our categories (2, 3) with cognitive, emotional, as well as practical benefits for both the parents and the child. However, in the themes of our study, we have focused on what underlies the perceived utilities and disutility of the test. In conclusion, knowledge was considered a key factor for making informed decisions for families.

### Scientific contribution and practical implication

The scientific contribution of this study is the knowledge that parents experienced genetic knowledge as an asset and that they wanted to understand why their child had SNHL. They experienced uncertainties as risks, but not the knowledge itself. There is a complexity in genetic investigations, and therefore providing information before and after testing is essential. Many parents in the study wanted more information than was provided. Thus, our conclusion is that additional clinical appointments with parents will be required, which is a time-consuming process in our clinical setting.

In the literature, barriers to performing genetic testing in children with hearing loss have been studied. Difficulties with insurance costs and lack of genetic knowledge were highlighted from both a parental (Cejas and Coto, 2024) and treating physician (Heyward et al., 2023) point of view. However, where health care is financed by the government, such as in Sweden, the financial limits are related to the healthcare provider and not the financial status of the parent. Given this, the clinician needs to be knowledgeable and propose testing only when it is relevant. A low probability of finding a genetic pathogenic variant, where there is no available genetic treatment option or low suspicion of a syndrome, are factors that can make testing less valuable for both parents and clinicians.

However, the practical implication of this study is that clinicians need not hesitate or postpone the testing procedure if there is a reasonable clinical gain. Based on the results of this study, parents are generally in favour of genetic testing, and they see personal, mental and practical benefits from various perspectives, and have an altruistic view of testing. On the other hand, parents who rejected testing were not included in our study. To complement the views on genetic testing for children with SNHL, it would be interesting to explore why parents refrained from testing their children.

The parental experience of the genetic investigation is, together with medical and ethical issues, important to consider when offering genetic testing to children with SNHL. The parental experience, in this field, is limited in research and this study complement the study by Tutty et al. (2021), which used open-ended questionnaires (Tutty et al., 2021). In this study, we investigated both the benefits and values, as well as the potential risks, associated with testing. The outcome of this study can support physicians in deciding when and whether to offer genetic testing to children with SNHL to ensure that care is in line with what parents (and the patients) need for a good care experience.

### Method discussion

Since one of the inclusion criteria was that the interviews should be in Swedish, there is a risk of cultural bias in the selected individuals. In our population, up to 40% of children with SNHL have parents originating from elsewhere in the world. Only two of the participating parents in this study were born outside of Sweden. Thus, generalisations for the whole population are questionable. Nevertheless, the argument, in favour of having Swedish spoken language as an inclusion criterion, was to avoid misinterpretations. This was considered important for the reliability of the study. The fact that two parents were born in other parts of the world means that the study did not reflect a solely Swedish cultural perspective. However, it would be interesting to include a more multicultural sample in future studies. To overcome the language barrier, professional translation could enhance inclusivity. However, care must be taken not to lose nuances in semantically meaningful units that might be lost in translation. Part of the meaning can be lost when using a translator, both in the questions to informants and their answers, affecting the reliability of the data.

In comparison with the study by Kohler et al. (2017), where they investigated personal and social outcomes with genetic testing, there are similarities in the sub categorises between their study and ours. Their themes (affective, cognitive, behavioural and social outcomes) are quite different, compared to ours, and are more prone to be topics. However, the personal and social outcomes they describe more or less reflect all subcategories in our themes 2 and 3. As they focused on personal utility, they did not describe anything corresponding to our first theme of uncertainty. However, the similarities in the sub-categories indicate that our findings are transferable to other settings.

As all the interviews and transcripts were conducted by the same person (JE), there was consistency during this process. The interviews were considered to contain rich data, and no obvious new categories were identified during the last interviews. However, as the study contains only nine interviews, there is a chance that complementing experiences and themes could be identified with additional participants. Nevertheless, both female and male participants are represented, as well as participants from different socioeconomical backgrounds and education levels. In this context, it must also be emphasised that this is a study where the parents have signed an informed consent form to perform the genetic test on their child. Thus, the experience of parents who do not want the genetic test to be performed on their child has not been explored. During the interview process, the material was read, coded and discussed by two researchers (JE, SW). In this sense, there was an inter-rater reliability when discussing codes and possible themes. Even though the researchers have different professions (otorhinolaryngologist and psychologist) and academic experience (PhD student and associate professor) there was an agreement on important meaningful quotes.

As both researchers are working in the field of audiology, preunderstanding and expertise could have influenced the analysis. However, this subjectivity can also be seen as an advantage, and the researchers acting as key instruments to grasp the content. In a reflexive manner, the initial themes were reviewed and redefined (Braun & Clarke, 2021). Once the themes were structured, the codes were re-checked and confirmed to ensure that the meaningful units were expressed in the basic, organising and global themes. What further increases credibility (Shenton, 2004) is the similarity of our results with the findings from Kohler et al. (2017).

## Conclusion and practical recommendations

Based on the parental experience, clinicians can be encouraged to offer genetic testing to children with SNHL, not only from a medical point of view, but also from a patient-centred perspective, where knowledge is considered important for the family and the future. Parents experienced that genetic testing and genetic knowledge were valuable on a personal level as well as for altruistic reasons. It was important for making the future predicable and had practical implications. However, to avoid anxiety, the parental need for information had to be met. The risk of vague genetic test results, as well as the uncertainty related to the future wishes of the child, could not be erased. Genetic knowledge could make decisions more complex, influence family planning, and was used to make predictions about the future. When offering genetic testing, it is advisable to provide written material and to offer genetic counselling. From the interviews, it was evident that parents had strong competence to handle the complex information from the genetic test. Thus, be confident that the parents can use the information to make decisions based on their needs.

## Data delivery statement

The original transcripts and codes in Swedish, supporting the findings of this study, are available upon request from the corresponding author. The data are not publicly available due to regulations on the ethical approval and could compromise the privacy of research participants.

## Supplementary Material

251028 Supplement 1.docx 251028 Supplement 1.docx
