# Supplementary material for: Parental experience of whole genome sequencing for children with sensorineural hearing loss
Source: Int J Qual Stud Health Well-being. 2026 Mar 12;21(1):2641802. doi: 10.1080/17482631.2026.2641802 (PMC12990280; doi:10.1080/17482631.2026.2641802)
Supplement: 251028 Supplement 1.docx [file ZQHW_A_2641802_SM6834.docx]

Supplement 1

Semi structured interview guide

HearSeq2: Qualitative descriptive interview study of how parents of children with moderate to severe hearing loss experience genetic testing with whole genome sequencing (WGS)

**Thank you for taking part in this study!**

**Introduction**

This is, as you probably already understood, an interview study. The interview is estimated to take between 45 minutes and one hour. We are doing this study to understand how parents of children with hearing loss who undergo genetic testing perceive the test and the genetic test result. Furthermore, we like to examine whether there are any perceived benefits or risks of the test. The interview will be recorded and analyzed and stored in our research department. If you have not already done so, you will be able to read the information before we start. If you want to participate, I need your written consent.

1. The genetic test result -present tense

- Do you know if your child has a genetic diagnosis?
- Can you tell me about what this diagnosis means?
- How do you feel about having a child with this diagnosis? Alternatively, how do you feel about your child not having a confirmed genetic diagnosis?
  - What are your thoughts/feelings about this?

1. Information – past tense

- When did you find out that your child has a hearing loss
- When was the genetic testing done?
- How did you get the information from the genetic testing?
- How did you perceive that information to be?
  - Why did you feel that way?
  - Can you give examples?
  - Would you like the information to be given in any other way?
- What benefit did you feel you got from the information when you received it?
  - In what way?
  - why not?
  - Do you know if the answer to the genetic test led to further action?

1. Follow-up – past tense

- Did you find out why your child has a hearing loss?
- How was it experienced?

1. Expectations – past in relation to present tense

- What were your expectations of what genetic testing would mean for your child?
- What were your thoughts before the test?
- How did you react to the results of the test?
- Were your expectations in line with what happened?

1. Emotions related to the genetic testing - past and present

- Did you have any emotional reaction to the result of the genetic testing?
- What are your feelings today about your child having a disability that may be hereditary?

1. Benefits and risks – present

- Do you feel that genetic testing is beneficial for you?
- Do you feel that there are risks associated with genetic testing?
- Can you give examples?
- Elaborate on your reasoning and how you think

1. Ethics – present
   - Do you perceive any the ethical aspects of genetic testing?
   - Did it influence your decision to participate in the study?
   - If you wish to have another child, will you/will you use the information obtained from the genetic testing?
2. Expectations for the future

- What do you think genetic testing could mean for your child in the future?
- What possibilities do you think this technology has in the future?
